# Welcome to Beautiful Mind; a Call to Action

**Published:** 2012

**Authors:** Fatemeh Heidary, Reza Gharebaghi

**Affiliations:** 1 School of Medicine, Shahid Beheshti University of Medical Sciences, Tehran, Iran; 2 Editorial Office, Medical Hypothesis, Discovery & Innovation Ophthalmology Journal

**Keywords:** Ophthalmology, Medical hypotheses, Innovation, Idea, Strategy

By definition, a hypothesis is a premise or assumption that is not yet verified or validated. The author looking to publish a bona fide hypothesis cannot do so by simply providing outline tables of raw data or pictures of tissues as proof (1). But, is now the time to start a journal in the field of ophthalmic hypotheses?

Unfortunately, visual impairment is a global and ubiquitous health problem. Thus far, there are a number of ophthalmology journals and hundreds of other journals that publish articles related to visual sciences to overcome the ocular problems and find novel solution and control of ophthalmic diseases as well as improve quality of life. Moreover, specialized ophthalmic hypotheses journal does not yet exist in the global forum. Although many scientific journals have special sections for publishing ideas and novel hypotheses, there appears to be an unoccupied niche for specialized journals like ours which has a wider scope. 

Medical Hypothesis, Discovery & Innovation (MEHDI) Ophthalmology Journal is an international peer-review journal in which we publish evidence-based ideas and hypotheses in the realm of ophthalmology and visual sciences. We look for discoveries, minimal invasive surgeries, new modalities of treatment, software, innovative instruments, and any texts that contain creativity and ideas in ophthalmology and visual sciences in order to publish them for maximum visibility. Journal has already been registered in well-known databases such as Index Copernicus, Genomics journal seek, Ulrich's, Research gate, Pubs Hub, RoMEO UK, and it will be soon registered in other reliable databases.

It is our belief that current and novel ideas deserve more credit; that they should be published in a timely manner, perhaps with some fanfare, if warranted. The idea being postulated must be argued in a clear and compelling manner at our journal. Reform movements always have some opposition therefore our journal provides space for critiques and publishes articles in a logical form in the ideology, and even for challenging ideas too. 

Usually, a researcher is concerned about others in making his discoveries and is therefore tremendously eager to have his ideas published as quickly as possible. Therefore, in our model, the average reviewing process would be 29 working days from submission to decision to respect the concern of scientists. Public Knowledge Project (PKP) management software administers publication in this journal and is one of the most reliable software packages in journal management. Many reliable scientific journals use that same software in order to improve the scholarly and public quality of academic research through the improvement of innovative online environments. This journal has several other interesting characteristics:

● Journal would also publish researches with no positive results as we believe that science is not merely about publishing positive outcomes. In some instances, if the researcher has negative results, these findings are seen as valuable so that another researcher does not waste his time conducting the same study. 

● Looking back on the current era, observers are unlikely to regard it as a ‘golden age’ of various developments in medical sciences (2) and majority of progress had been occurred in science and technology. The history of science and progress of humanity is of great importance and understanding it would be a most positive direction for moving forward. Therefore, historical articles of science or philosophy of science should receive a significant amount of attention in this journal.

● Sometimes, we publish innovative laboratory processes and techniques in testing in order to improve the methodology and design of future studies. 

● It is important to consider new ideas from a broader perspective; that we shouldn’t necessarily limit ourselves to the current content in laboratories. For instance, a researcher might perform extensive research in molecular sciences on a special enzyme, gene or a biochemical pathway and ignore the social determinants of diseases. We believe that it is critically important to widen the field of ideas from molecular to population-based studies. In this case, with all due respect to molecular sciences, our journal anticipates ideas and hypotheses that focus on social and psychological issues as well.

● Journal proves that publication of similar specialty and subspecialty areas are required. A team is working to establish the feasibility of journals with same goal in optometry and seven sub specialized sections.

● As of now, this journal tries to classify and publish subspecialty papers. In this volume, major articles which have been published are in the anterior segment section and in future issues we will work subspecialties on the retina, glaucoma, basic science, etc. 

● Sometimes, other journals do not allow for the publishing of new ideas and hypotheses. They ask for evidence and up-to-date references for even small sentences. In our journal, ideas with merit are published immediately if they are new, practical, and well described. 

● In some issues, due to the importance of some of the subjects, we publish invited papers. As an example, the pervasiveness of poverty throughout the world has meant that many diseases cannot be controlled. For that reason, we invite researchers who have ideas on how to best solve or mitigate against this universal crisis to submit their manuscripts to the journal.

● This journal attributes high importance to the exchanging of information and discussions in the published papers as there might be new ideas emerging from those discussions, and those ideas could be a causal factor in future positive change. Ideas from other researchers across the world are a welcome contribution.

As stated, we are fan of new ideas; however, the opinions that are published in this journal are solely those of the authors. The publication of them does not mean full approval of their hypotheses; nor are they the opinion of the editorial board and Editors-in-Chiefs. The validity and vigorousness of references at the end of articles are also considered as we hope to provide a good forum for authors to source new ideas.

Essentially, hypotheses are not necessarily theoretical issues. There might be cases when a physician observes an unknown disease or syndrome in his clinic and if the findings are remarkable enough, he might submit them in a manuscript. If the paper has been prepared in a logical manner and there is evidence based on scientific fact, we would publish it in our journal immediately. Our emphasis is on high quality articles which we would have journal-indexed in distinguished databases immediately so that they would have maximum visibility. The journal also publishes ideas that might have only a theoretical basis. In addition, if a researcher tests the idea published in the journal in a scientific framework, and submits his findings to the journal based on the fact presented at our former issues, that paper would be immediately published as well.

Articles that do not have fresh ideas or were not prepared logically, and/or were not able to convey their meaning to the reader would not be published. At the same time, this journal is highly sensitive to science misconduct and if there are indications of fraud and plagiarism in a paper, it would not be published and/or simply retracted. Our goal is that after reading the paper, new ideas are formed in the reader’s mind which in turn will generate positive changes in a honestly situation. Perhaps, at the end of each year, the accumulation of applied ideas could be published as a book, such as “Death Can be Cured” which was a product of papers published in Medical Hypothesis (3).

We believe that, if only one scientific change could be achieved, or a major medical problem solved from the hundreds of ideas and hypotheses that will be published in the journal, the editorial team would feel that they have made a noteworthy contribution.

The editorial board of this journal includes elected university professors, lecturers, and researchers who have already published valuable scientific studies. As evident in their titles and affiliations, the board has been selected from five continents and a number of the members are presidents of prestigious ophthalmology societies from all around the world. And finally, our major goal is to push ophthalmology forward by presenting innovative ideas and discoveries. On behalf of the editorial team, we welcome potential subscripts and we hope our presence in the variety of scientific journals would build the groundwork toward new changes in life, health and medicine. It is our hope that the papers prove to be valuable; certainly worth the time it takes to read them.

## DISCLOSURE

The authors report no conflicts of interest in this work.
